# What is the impact of a novel MED12 variant on syndromic conotruncal heart defects? Analysis of case report on two male sibs

**DOI:** 10.1186/s13052-020-00865-w

**Published:** 2020-07-18

**Authors:** Silvia Amodeo, Giuseppe Vitrano, Melania Guardino, Giuseppe Paci, Fulvio Corselli, Vincenzo Antona, Giuseppe Barrano, Monia Magliozzi, Antonio Novelli, Renato Venezia, Giovanni Corsello

**Affiliations:** 1 Department of Obstetrics and Gynecology, University Hospital Policlinico P. Giaccone, Via Alfonso Giordano 3, Palermo, Italy; 2 Department of Neonatology and NICU, University Hospital Policlinico P. Giaccone, Palermo, Italy; 3 San Pietro Fatebenefratelli Hospital, UOSD Medical Genetics, Rome, Italy; 4 Bambino Gesù Children’s Hospital, Laboratory of Medical Genetics, Rome, Italy

**Keywords:** Congenital heart diseases, Conotruncal heart defects, Facial dysmorphisms, MED12, Echocardiography, Next generation sequencing, Case report

## Abstract

**Background:**

Syndromic congenital heart disease accounts for 30% of cases and can be determined by genetic, environmental or multifactorial causes. In many cases the etiology remains uncertain. Many known genes are responsible for specific morphopathogenetic mechanisms during the development of the heart whose alteration can determine specific phenotypes of cardiac malformations.

**Case presentation:**

We report on two cases of association of conotruncal heart defect with facial dysmorphisms in sibs. In both cases the malformations’ identification occurred by ultrasound in the prenatal period. It was followed by prenatal invasive diagnosis. The genetic analysis revealed no rearrangements in Array-CGH test, while gene panel sequencing identified a new hemizygous variant of uncertain significance (c.887G > A; p.Arg296Gln) in the MED12 gene, located on the X chromosome and inherited from the healthy mother.

**Conclusion:**

No other reports about the involvement of MED12 gene in syndromic conotruncal heart defects are actually available from the literature and the international genomic databases. This novel variant is a likely pathogenic variant of uncertain significance and it could broaden the spectrum of genes involved in the development of congenital heart diseases and the phenotypic range of MED12-related disorders.

## Background

Conotruncal heart defects (CTDs), account for approximately 30% of congenital heart disease (CHDs). They are characterized by anomalies of the cardiac outflow tract or of the great arteries [1]. These include common arterial trunk (CAT) and aorto-pulmonary window (APW), as well as double outlet right ventricle (DORV), tetralogy of Fallot (TOF) with or without pulmonary atresia (PA), transposition of the great arteries (TGA), interrupted aortic arch (IAA).

CAT is characterized by a single great artery arising from the ventricles, giving origin to aorta, coronary arteries and pulmonary arteries, and by a ventricular septal defect (VSD) [2]. According to the Collett and Edwards’ Classification [3], CAT is distinguished in: type I when a single pulmonary trunk arises from the truncus arteriosus, type II when two pulmonary branches arise separated but close from the posterior wall of the truncus arteriosus, type III when two pulmonary branches arise separated from lateral walls of the truncus arteriosus. Type IV now is defined pulmonary atresia with ventricular septal defect (PAVSD).

Aorto-pulmonary window (APW) is a communication between the ascending aorta and the pulmonary artery in the presence of separate semilunar valves [4]. It was classified by Richardson into three types [5]: in type I the defect between the aorta and the main pulmonary artery is immediately above the sinuses of Valsalva, in type II the defect is located more distally between the ascending aorta and the origin of the right pulmonary artery and in type III there is anomalous origin of the right pulmonary artery from the ascending aorta.

Both CAT and APW are determined by the failure of septation of the physiologic arterial common trunk into aorta and pulmonary artery trunk during development of the ventricular outlets and proximal arterial segment of the heart tube.

We report on a novel variant in the MED12 gene, detected by Next Generation Sequencing (NGS), associated with recurrent conotruncal heart defects, CAT and APW respectively, and facial dysmorphisms in an Italian family.

## Case presentation

### Case 1

A couple, 33 year-old woman and 34 years-old man, referred to Prenatal Diagnostic Unit of Policlinico Universitario Paolo Giaccone for a routine mid-trimester ultrasound scan of their first pregnancy. Conception was spontaneous, there was no consanguinity and the couple was in excellent health without any medical history. They had decided to not perform the first trimester screening for fetal aneuploidy.

**Fig. 1:**
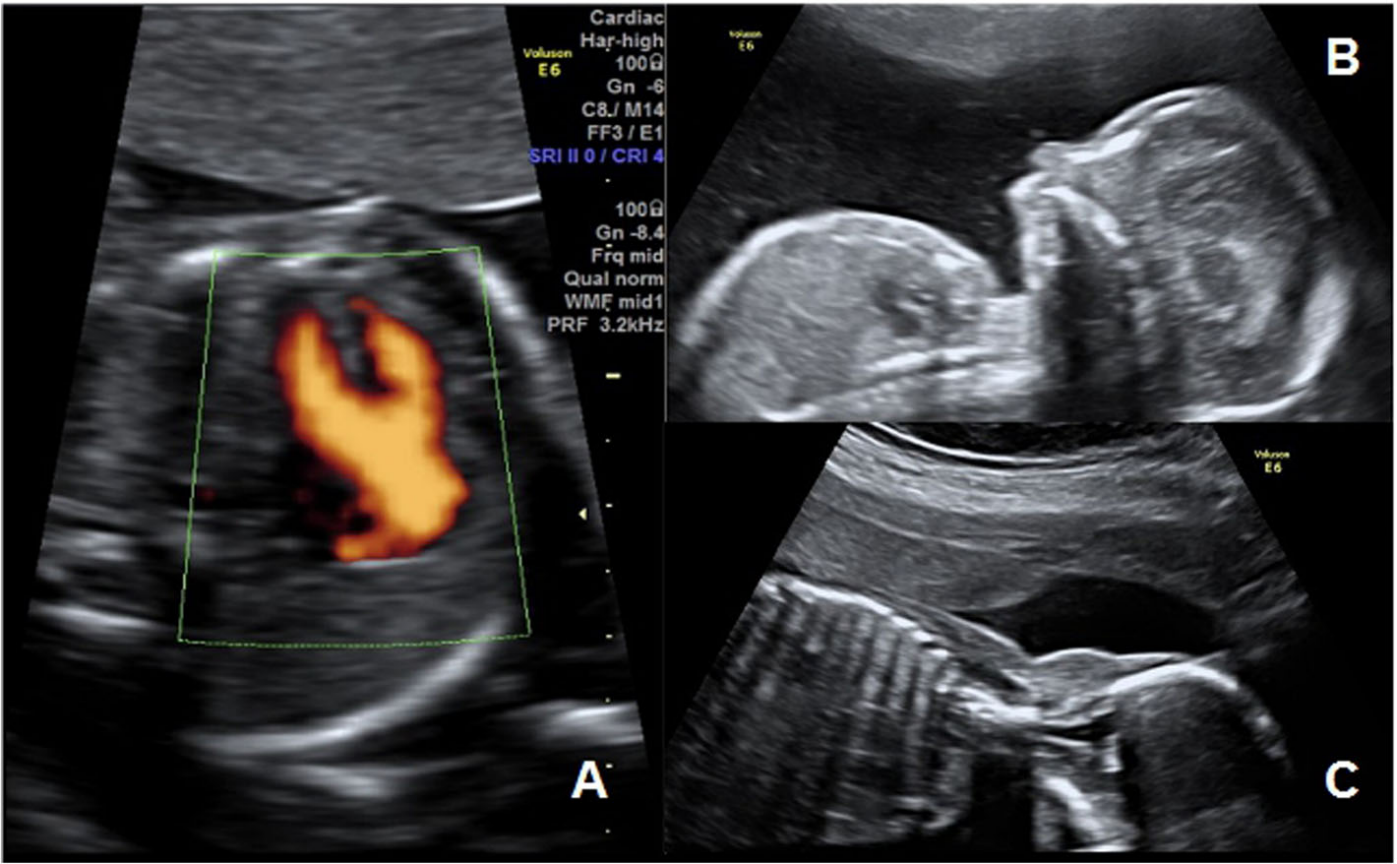
Case 1 mid-trimester ultrasound scan. **a** single great artery that equal overrode a ventricular septal defect; **b** microretrognathia; **c** nuchal edema

The mid-trimester ultrasound scan, performed at 20.1 weeks of gestation, showed a male fetus with normal four-chamber view but abnormal outflow tract views: a single great artery equal overrode a ventricular septal defect (VSD) (Fig. 1a). The main pulmonary trunk and subsequent bifurcation arose from the single great artery immediately distal to the truncal valve. Color Doppler confirmed the blood flow through the VSD, the overriding artery, the origin of the pulmonary artery and its bifurcation. This finding suggested a diagnosis of truncus arteriosus type I with VSD according to the Collett and Edwards’ Classification [3]. Ultrasound research of associated extra-cardiac abnormalities has highlighted microretrognathia and nuchal edema (Fig. 1b and c).

CAT is often associated with extra-cardiac anomalies in the context of specific syndromes, the most common is 22q11 microdeletion [2], so, following genetic counseling, amniocentesis for karyotype and Array-CGH (array comparative genomic hybridization) was performed. The Array-CGH was performed with CytoSure ISCA v2 Arrays 8x60k. Both the standard karyotype and the CGH-arrays were negative (arr (1–22) × 2,(XY)× 1).

The couple opted for the voluntary termination of the pregnancy and, on the advice of geneticists, requested to store fetal DNA.

### Case 2

**Fig. 2:**
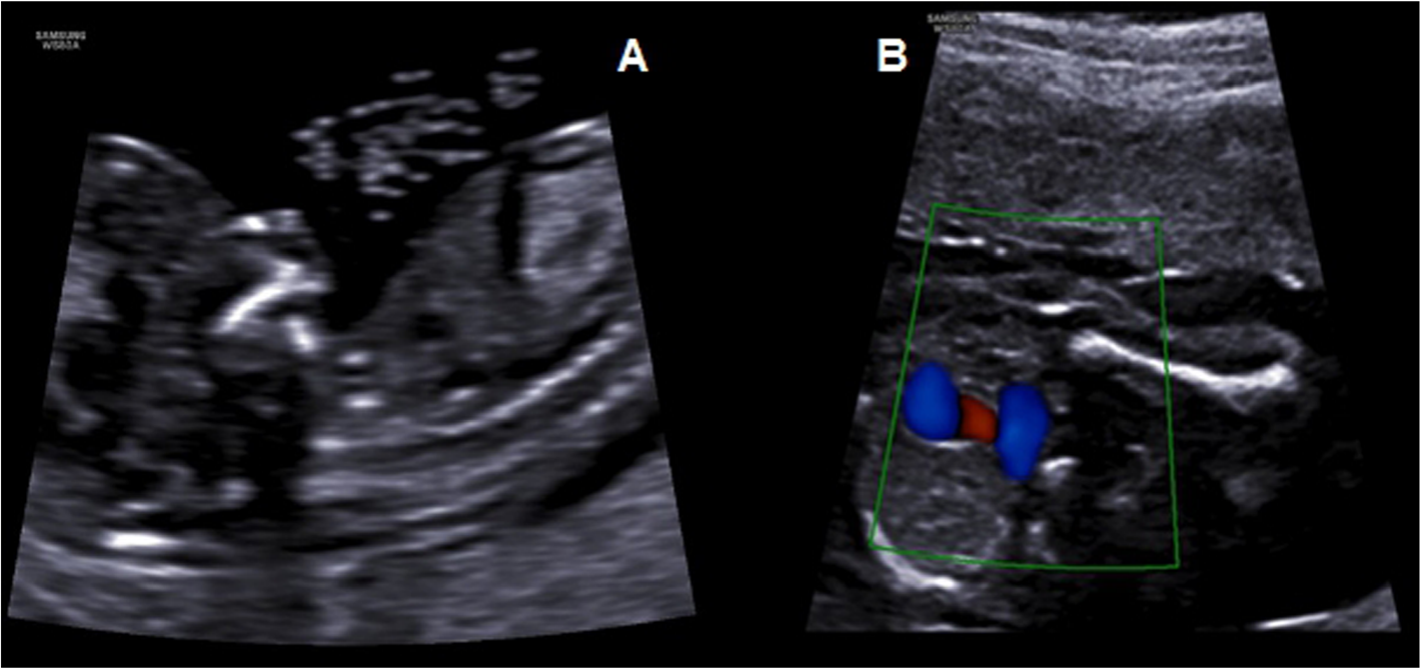
Case 2 ultrasound finding. **a** first-trimester ultrasound scan shows microretrognathia; **b** three vessels and trachea view shows the communication between aorta and pulmonary artery (aorto-pulmonary window)

One year later, the same couple referred again to our Prenatal Diagnostic Unit for the second pregnancy at 12.5 weeks of gestation for first trimester combined screening for fetal aneuploidy. Free β-hCG and PAPP-A were 1.397 MoM and 1.55 MoM. Nuchal translucency was 1.4 mm, nasal bone present, FHR 159 bpm, ductus venosus PI 1.15 and tricuspid regurgitation and microretrognathia were detected (Fig. 2a). Ultrasound showed no other fetal anomalies evaluable at the first trimester. Patient-specific risks for trisomy 21, 18, 13, computed based on the FMF algorithm, were 1/350, 1/1862 and < 1/20.000 respectively. Considering the tricuspid regurgitation and microretrognathia and the history of fetal malformation in the previous pregnancy, following genetic counseling, the couple declined villocentesis via karyotype and microarray analysis. Therefore, early fetal echocardiography was performed at 18 weeks: three vessels and trachea view showed a communication between aorta and pulmonary artery immediately above the sinuses of Valsalva, so an aorto-pulmonary window type I according to Richardson’s Classification [5] (Fig. 2b) and thymic hypoplasia. A detailed ultrasound to assess fetal anatomy confirmed the microretrognathia in male fetus. Therefore, following genetic counseling, the couple accepted amniocentesis for karyotype and Array-CGH. The Array-CGH was performed with SurePrint G3 Human CGH Microarray 8x60k. Again, both the standard karyotype and the Array-CGH were negative (arr (1–22) × 2,(XY)× 1) and the couple decided to the voluntary termination of the pregnancy and to store fetal DNA.

**Fig. 3:**
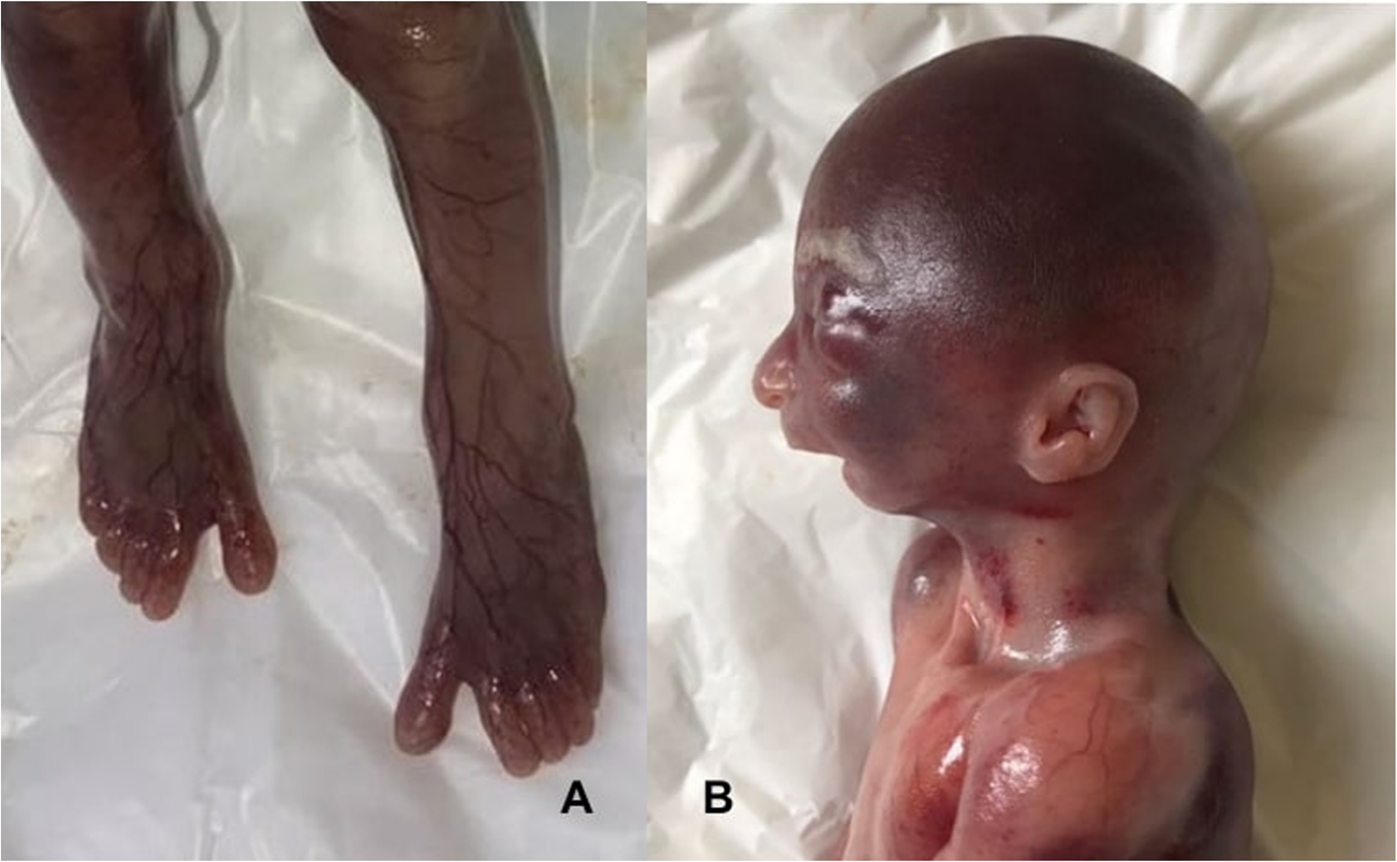
Autopsy findings.: **a** sandal gap from first case; **b** microretrognathia, small mouth and low-set ears from second case

The autopsy of aborted fetuses confirmed in both male sibs the conotruncal heart defects, truncus arteriosus in the first fetus and aorto-pulmonary window in the second, the same facial dysmorphisms (microretrognathia, small mouth, low-set ears) and sandal gap (Fig. 3).

Considering the recurrence of the association of conotruncal hearts disease and facial anomalies in both sibs, obtained informed consent for genetic testing, molecular genetic study was performed on genomic fetal DNA extracted from amniotic fluid through Next Generation Sequencing (NGS), evaluating cardiopathy-associated genes (Table 1). We used targeted resequencing with a uniquely customized design SeqCap EZ Custom Enrichment Kit (Roche Life Science) on Illumina sequencing platform (NextSeq550, San Diego, CA). We used the BaseSpace pipeline (Illumina, https://basespace.illumina.com/) and the VariantStudio software (Illumina, http://variantstudio.software.illumina.com/) for the variant calling and annotating variants, respectively. Sequencing data were aligned to the hg19 human reference genome. We analyzed the variants in silico by using Scale-Invariant Feature Transform (SIFT), Polymorphism Phenotyping v2 (PolyPhen-2) for the prediction of deleterious non-synonymous SNVs for human diseases. The analysis revealed the hemizygous variant, NM_005120.2: c.887G > A (p.Arg296Gln) in MED12 gene located on the X chromosome. Sanger sequencing following a standard protocol (BigDye Terminator v3.1 Cycle Sequencing Kit, Applied Biosystems by Life Technologies) confirmed the variant and we tested it for familial segregation using the maternal DNA and the fetal DNA from the first pregnancy showing the heterozygous and hemizygous state respectively. This finding may explain the fetal phenotype and X-linked trasmission. The whole exome sequencing (WES) was not performed in relation to the diagnostic relevance of the genetic results.

**Table 1:** Cardiopathy-associated genes evalueted

ACTA2	CFC1	FBN2	GDF2	MYH11	RASA1	TGFB2
ARHGAP31	CRELD1	FBN1	GATA4	MYLK	RBM10	TGFB3
ACVR2B	CITED2	FMN1	GDF1	MYH6	RBPJ	TGFBR1
ACVRL1	COL3A1	FMN2	HAND2	MED12	SALL4	TGFBR2
ADAMTSL4	DOCK6	FOXC1	HAND1	MED12L	SLC2A10	TGFBR3
BMPR1B/ALK6	DLL4	FOXH1	HEY2	NODAL	SMAD3	TOPBP1
BCOR	DIAPH2	FHOD1	ISL1	NFATC1	SOX7	TFAP2B
bcl9l	DNAH11	FGF8	LEFTY1	NKX2–5	SKI	TBX1
BMP4	DNAH5	FHOD3	LEFTY2	NAT10	SMAD4	TBX2
BMPR2	DIAPH1	FOXA2	JAM3	NKX2–6	SMAD1	TBX4
B3GAT3	EIF2AK4	FOXF1	JAG1	NOTCH1	SMAD6	TBX20
CRKL	ELN	FLNA	KCNK3	NOTCH2	SMAD9	TBX3
CBLN2	EFEMP2	GDF1	MCTP2	NOTCH3	SALL1	ZIC3
CBS	ENG	GJA1	MID1	PITX2	TLL1	ZFPM2/FOG2
CAV1	ETS-1	GJA5	MAPK1	PPARG	SHROOM3	ZDHHC9
CFAP53	EOGT	GATA6	MHC6	PLOD1	TGFB1	

## Discussion and conclusions

The prenatal finding of recurrent CTDs, truncus arteriosus and aorto-pulmonary window respectively, associated with facial dysmorphisms in an Italian family gave us the suspicious of a syndromic CHDs¸ inducing the search for a possible genetic cause.

Congenital heart defects (CHDs) are structural defects due to abnormal cardiac development in embryos. Approximately 70% of CHDs present as an isolated malformation (non-syndromic CHD), while 30% are syndromic because in association with other extra-cardiac anomalies [6]. Causes of CHDs are genetic in 8%, environmental in 2% and multifactorial or not determined in about 90% of cases [7]. The genetic mechanisms responsible for CHD are complex, heterogeneous and incompletely understood: approximately 8–12% is due to chromosomal anomalies, 3–25% to copy number variation and 3–5% to single gene defect [8]. These genetic variants, de novo or inherited, concern genes involved in normal cardiac development, altering signal transduction, transcriptional regulation and encoding proteins. In some cases the same genes have pleiotropic effects on other organs [8] and this explains syndromic CHDs.

Clark EB [9] classified the CHDs according to the pathogenesis: conotruncal defects are classified among the anomalies of the migration of the ectomesenchyme, in which there is an anomalous migration of component of the secondary/anterior heart field. During heart development the outflow tract develops by adding cells from the pharyngeal mesoderm called the secondary/anterior cardiac field [10], whereas the aorto-pulmonary septum develops by condensation of cardiac neural crest (CNC) cell – derived mesenchyme [11]. Alteration or loss of these developmental processes leads to conotruncal anomalies. The increasing studies of the genetic etiology of CHDs allowed the identification of many genes involved in specific morphogenetic mechanisms and responsible of specific cardiac phenotype, creating a link between cause, pathogenetic mechanism and type of malformation [12]. Conotruncal heart diseases are associated with various chromosomal pathologies and monogenic syndromes: the most frequent associations of the CAT are with DiGeorge syndrome, interstitial duplication of 8q and CHARGE syndrome, whereas the APW can be associated to VACTERL association and CHARGE syndrome [13]. Among the genetic variants associated with conotruncal defects, the MED12 gene does not currently appear. MED12 gene is located on chromosome X and encodes a subunit of the Mediator Complex, called mediator complex subunit 12, which is crucial in RNA polymerase II transcription [14]. Zebrafish models have shown that Med12, being co-regulator of specific transcription factors, is necessary during development: med12 zebrafish mutants showed defects in neural crest formation, chondrogenesis and organogenesis of the brain, liver, pancreas and kidney [15, 16]. Zebrafish and mouse MED12-mutant demonstrated the involvement of the MED12 gene in heart development [17, 18].

Our study reports for the first time the finding of variant in the MED12 gene associated with syndromic CHD, specifically associated with facial dysmorphisms. A probable explanation of the role of the MED12 gene in determining conotruncal defect would be found in the molecular mechanisms involved in embryonic development of the heart: Med12 regulates gene-specific functions during development and it is necessary for correct Wnt/β-catenin signaling and Wnt/planar cell polarity (PCP) pathway, as demonstrated on mouse models by Rocha et al. [18] The consequent alteration of the Wnt pathway and of the PCP signaling would therefore be responsible for the lack of migration of the CNC cells during the formation of the aorto-pulmonary septum. Schleiffarth et al [19] have indeed shown that the Wnt signal, in particular Wnt5a, is necessary for the correct development of the aorto-pulmonary septum and that its loss is responsible for the development of conotruncal defects.

The variant reported in our study is not described in the international literature (PubMed/Medline) and in the Catalog of Human Genes and Genetic Disorders (OMIM) and it is currently to be considered VOUS (Variants Of Uncertain Significance). The normal phenotype of the mother carrying the variant on the X chromosome in heterozygosity and the recurrence of the pathological phenotype in fetuses both male with hemizygous state increase the suspicion that this variant is the cause of the pathology in these family patients and that it may explain an X-linked transmission of syndromic conotruncal defects, contributing to increase the phenotypic range of MED12-related disorders. Therefore it will have important implications for genetic and preconception counseling, family planning and clinical management.

In conclusion, despite we know many genetic variations involved in the development of CHDs and specifically of CTDs, in most patients the genetic defects remain unknown [1]. The latest investigation techniques such as next generation sequencing (NGS) could increase our knowledge about CHD genetic etiology. The variant c.887G > A (p.Arg296Gln) in MED12 gene is a likely pathogenic VOUS and it could shed new light on the possible pathogenesis of conotruncal heart defects.

## Data Availability

All the data presented in this article are stored in Prenatal Diagnostic Unit of University Hospital Policlinico Paolo Giaccone.

## Abbreviations

CTDs: ConoTruncal defects
CHDs: Congenital heart disease
CAT: Common arterial trunk
APW: Aorto-pulmonary window
DORV: Double outlet right ventricle
TOF: Tetralogy of Fallot
PA: Pulmonary atresia
TGA: Transposition great arteries
IAA: Interrupted aortic arch
VSD: Ventricular septal defect
PAVSD: Pulmonary atresia with ventricular septal defect
NGS: Next Generation Sequencing
Array-CGH: Array comparative genomic hybridization
VOUS: Variants Of Uncertain Significance
